# Mechanisms of the biological response cascade to exercise-induced stress: a comprehensive review

**DOI:** 10.3389/fspor.2025.1691779

**Published:** 2025-11-13

**Authors:** Jing Xu, Junjun Zhang, Kai Sang

**Affiliations:** 1 Department of Police Tactics, Fujian Police College, Fuzhou, China; 2 Fujian Key Laboratory of Innate Immune Biology, Biomedical Research Center of South China, College of Life Sciences, Fujian Normal University Qishan Campus, Fuzhou, Fujian Province, China; 3 China Institute of Sport Science, Beijing, China

**Keywords:** exercise-induced stress, bidirectional threshold, biological response cascade, adaptation and maladaptation, exercise variability

## Abstract

Exercise is widely recognized as a critical determinant of health, yet its effects can diverge substantially depending on intensity, duration, and individual characteristics. This review synthesizes current knowledge on the mechanisms underlying exercise-induced stress responses, outlining a sequential cascade from biomechanical signal perception, through organelle and metabolic regulation, to systemic integration via hormonal, myokine, and immune pathways. We highlight the concept of a bidirectional threshold theory, which proposes that moderate exercise promotes adaptation and health benefits, while excessive exercise may trigger maladaptive responses and pathological outcomes. At the same time, we note that significant inter-individual variability in exercise responses raises important questions regarding the generalizability of this framework. By integrating evidence across molecular, cellular, and systemic levels, this review provides a holistic perspective on the dual effects of exercise, underscores the need for improved biomarkers to monitor adaptive vs. maladaptive responses, and identifies research gaps that must be addressed to translate these mechanisms into personalized exercise strategies.

## 1 Introduction

Exercise is an essential component of human health, and its physiological impact has long been a central focus of biomedical research. With advancements in exercise biology, researchers have increasingly elucidated the profound effects of exercise on cellular stress responses, tissue remodeling, and systemic regulation (1–4). Beyond its well-documented role in enhancing physical performance, exercise also exerts significant influence on mental well-being, serving as a non-pharmacological strategy to alleviate psychological stress and reduce the incidence of anxiety and depression (5, 6). Understanding the biological response mechanisms associated with exercise-induced stress is therefore crucial for the rational application of exercise in health promotion and disease prevention.

The stress response elicited by exercise is a complex process involving cardiovascular adaptation, endocrine modulation, immune activation, and intracellular signaling cascades (7). These responses exhibit clear dose-dependent effects. In this context, the bidirectional threshold theory has emerged as a valuable framework: moderate exercise elicits beneficial adaptations, whereas excessive exercise can lead to pathological damage depending on the intensity and duration of the stimulus (8). For example, the production of reactive oxygen species (ROS) during moderate exercise activates adaptive signaling pathways (e.g., JNK, NF-κB), thereby enhancing antioxidant defenses and repair mechanisms (9, 10). Conversely, chronic ROS overload may trigger apoptosis, mitochondrial dysfunction, and pro-inflammatory signaling, ultimately accelerating tissue damage (10). Similarly, while mechanical loading stimulates integrin-FAK-Akt signaling to promote cell survival and differentiation, sustained or excessive activation may predispose tissues to fibrosis and maladaptive remodeling (11–14). Despite these advances, current literature is often fragmented, with a stronger emphasis on adaptive outcomes while insufficiently addressing maladaptive or pathological responses to overtraining. This imbalance hinders a comprehensive understanding of the “duality” of exercise. Moreover, many studies rely on cross-sectional or animal models, leaving gaps in longitudinal human data that could validate molecular findings in real-world training scenarios (15). Individual differences—including sex, age, genetic background, and comorbidities—further complicate the interpretation of exercise responses, yet remain underexplored (16). These limitations highlight the need for a more critical and integrative perspective that connects molecular mechanisms with clinical translation (17, 18).

To address these gaps, this review synthesizes recent findings into a unified conceptual framework of the biological response cascade to exercise-induced stress. Specifically, it progresses through three interconnected layers: (i) primary responses, including mechanical signal transduction and organelle stress; (ii) secondary regulation, emphasizing metabolic reprogramming and energy sensing; and (iii) systemic integration, involving endocrine, immune, and neuro-metabolic networks.

Within this framework, we highlight both the adaptive and maladaptive trajectories of exercise responses, aiming to establish a balanced understanding of how exercise can act as both a health-promoting stimulus and a potential pathological challenge. By critically analyzing existing evidence, identifying research limitations, and proposing future directions, this review seeks to provide a more comprehensive foundation for personalized exercise prescriptions and translational exercise medicine.

## 2 Primary responses to exercise-induced stress signals

**Figure 1:**
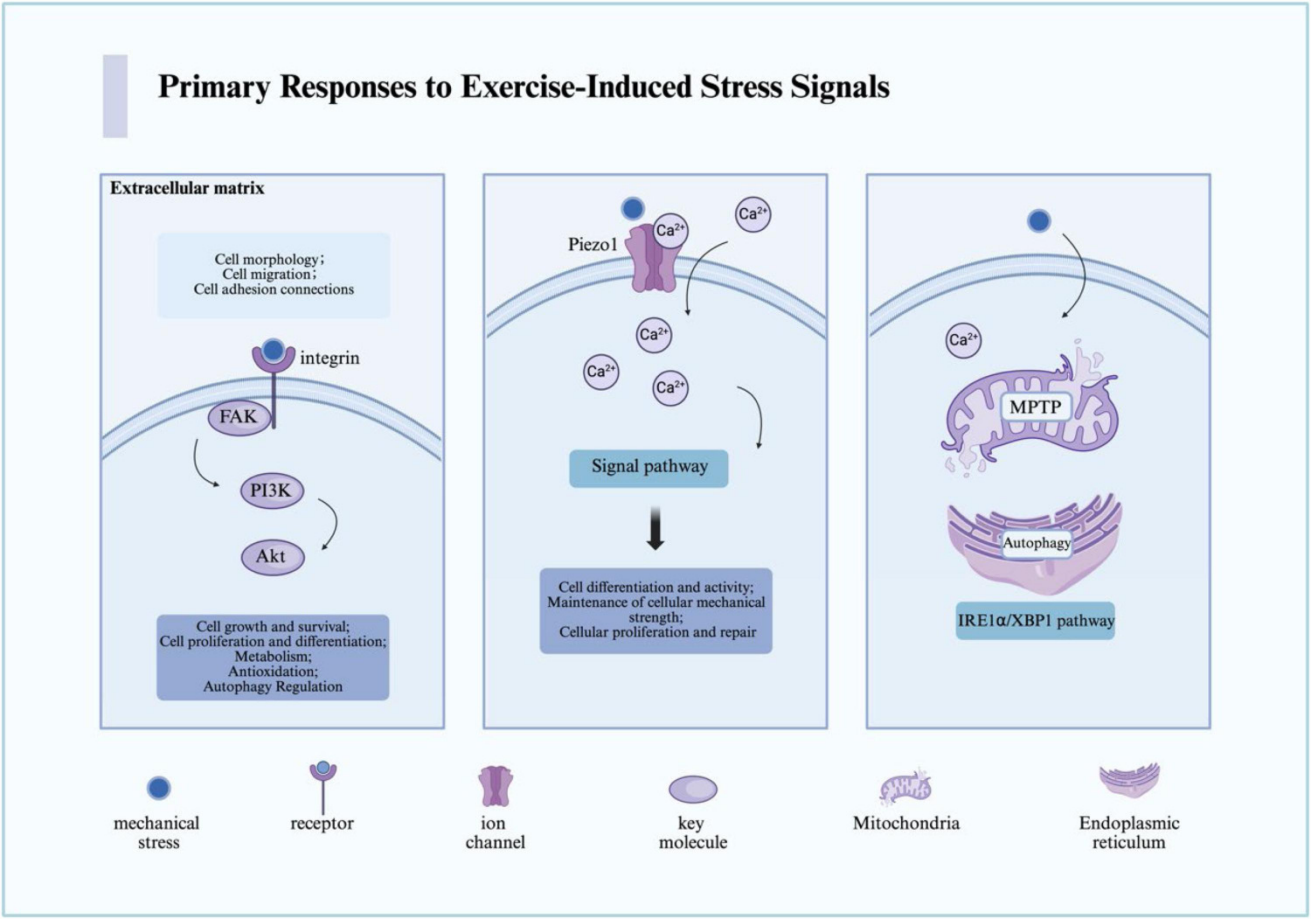
The primary pathways through which cells sense mechanical stress derived from exercise-induced stress are illustrated. In the figure, we present the key receptors, pathways, and cellular responses involved in the sensing and transduction of mechanical stress. For detailed descriptions, refer to the corresponding sections in the text. This figure was created using Biorender. MPTP, mitochondrial permeability transition pore; Ca, calcium; FAK, focal adhesion kinase; PI3K, phosphoinositide 3-kinase; Akt, also known as protein kinase B (PKB); Piezo1, piezo-type mechanosensitive ion channel component 1.

Exercise triggers a diverse array of primary stress signals at the cellular level, which are first sensed through mechanical transduction and organelle responses (19, 20). These mechanisms represent the foundation of the adaptive cascade but also constitute the initial nodes where maladaptation may arise under conditions of excessive or prolonged stimulation (21, 22) (Figure 1).

### 2.1 Mechanical signal transduction

Cells perceive external mechanical forces primarily through integrins, focal adhesion kinase (FAK), and mechanosensitive ion channels such as Piezo1. Under moderate loading, integrins activate FAK and downstream PI3K/Akt signaling, which promote survival, proliferation, and differentiation—key processes for tissue adaptation and repair (11, 14, 23). Similarly, Piezo1-mediated Ca^2+^ influx enhances osteoblast differentiation and vascular remodeling, supporting musculoskeletal and cardiovascular health (24–26).

However, the same pathways can contribute to pathological remodeling when excessively or persistently activated. Continuous Piezo1 activation may induce pathological Ca^2+^ overload, leading to mitochondrial dysfunction, inflammasome activation, and maladaptive fibrosis (27–29). Similarly, sustained FAK overexpression has been implicated in fibrosis and tumor progression, suggesting that while transient activation supports regeneration, chronic overstimulation may shift toward disease phenotypes (30, 31). These dual effects exemplify the bidirectional threshold theory, emphasizing that the biological outcome depends not only on whether these pathways are activated but also on the intensity, duration, and recovery dynamics of the stimulus (32).

The threshold for activation also differs by exercise mode. For example, endurance training typically induces moderate, repetitive integrin-FAK-Akt activation that supports angiogenesis and mitochondrial biogenesis (33), whereas resistance training imposes acute high-intensity loads that more strongly activate mTORC1-mediated anabolic pathways (34–36). Yet, excessive resistance training may surpass the adaptive threshold, leading to inflammatory microdamage and impaired recovery (37). Future research should quantify these thresholds across exercise types to define the molecular boundaries between adaptation and overtraining injury (38).

Quantitatively, in many human studies IL-6 levels have been observed to increase several-fold (e.g., ∼5-fold) within 1–3 h after a bout of endurance exercise (39). Similarly, serum BDNF concentrations are commonly reported to rise substantially (e.g., tens of percent) in the first hour following moderate exercise (40, 41). More precise quantification across various exercise modalities and populations remains a priority for future work.

### 2.2 Interactions between organelles

Mitochondria and the endoplasmic reticulum (ER) serve as critical hubs of cellular stress responses (42, 43). Moderate exercise enhances mitochondrial oxidative capacity and transiently activates the unfolded protein response (UPR), supporting proteostasis and energy supply (44). However, under excessive exercise, pathological events emerge: persistent opening of the mitochondrial permeability transition pore (MPTP) can trigger ATP depletion and apoptosis, while unresolved ER stress can shift from adaptive UPR to pro-apoptotic signaling (via CHOP and JNK), culminating in cell death (45, 46).

Importantly, mitochondria and the ER are not isolated. They communicate through specialized structures known as mitochondria-associated membranes (MAMs), which mediate Ca^2+^ flux, ROS signaling, and lipid transfer (47). Exercise modulates these interactions in a bidirectional manner (43). Moderate exercise promotes Ca^2+^-dependent mitochondrial activation and metabolic efficiency, whereas excessive Ca^2+^ transfer via MAMs may lead to mitochondrial Ca^2+^ overload, ROS accumulation, and apoptotic signaling (48). In addition, lysosomes also participate in this crosstalk by regulating autophagy and mitophagy, processes essential for clearing damaged organelles and maintaining cellular homeostasis (49, 50). Dysregulation of these networks under chronic overtraining may therefore contribute to systemic fatigue and impaired recovery (50, 51).

In summary, primary stress responses to exercise involve finely tuned signaling through mechanical sensors and organelle networks. While these pathways underpin the health benefits of physical activity, their chronic or excessive activation can drive maladaptive remodeling and disease. This duality underscores the importance of exercise “dose” and sets the stage for secondary metabolic reprogramming, discussed in the following section.

## 3 Secondary regulation of exercise-induced metabolism

**Figure 2:**
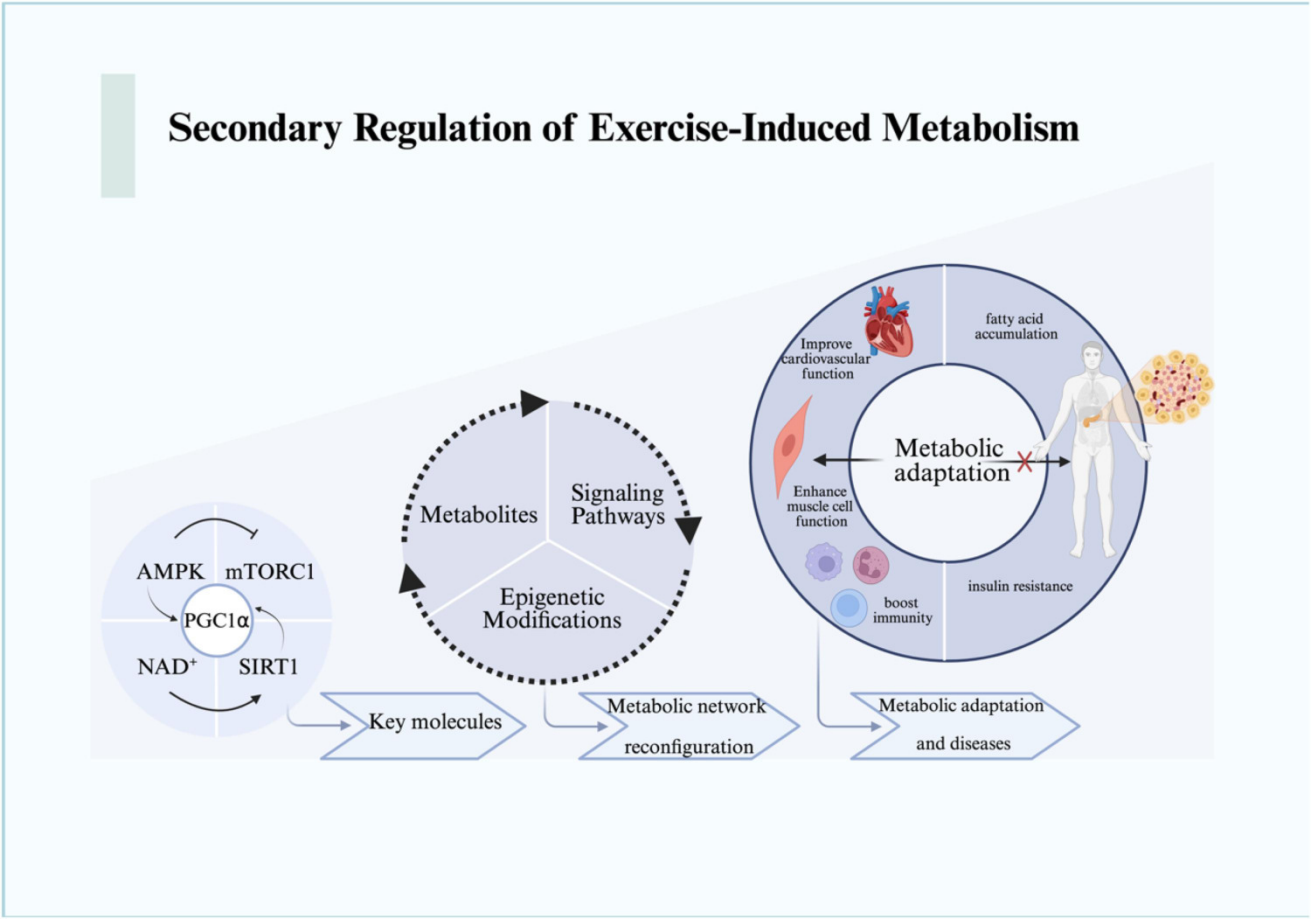
The metabolic responses and changes resulting from the mechanical stress signal transduction cascade are illustrated. We depict the metabolic reactions that expand from the molecular level to the cellular level and beyond, encompassing the macro processes of metabolic remodeling and adaptation. For detailed descriptions, refer to the corresponding sections in the text. This figure was created using Biorender. AMPK, AMP-activated protein kinase; mTORC1, mammalian target of rapamycin complex 1; NAD^+^, nicotinamide adenine dinucleotide; PGC1α, peroxisome proliferator-activated receptor gamma coactivator 1-alpha; SIRT1, sirtuin 1.

This cascade can be temporally framed: mechanical signals emerge within seconds to minutes, metabolic and organelle adaptations occur over hours, while systemic endocrine and immune effects manifest across days to weeks (52). Beyond the primary mechanical and organelle-level responses, exercise triggers profound changes in cellular metabolism (53). These secondary regulatory mechanisms revolve around the cell's capacity to sense and respond to energy fluctuations, reprogram metabolic networks, and establish long-term adaptations through epigenetic regulation (Figure 2).

### 3.1 Energy sensing: AMPK, mTORC1, and NAD^+^

Energy sensing is primarily mediated by AMP-activated protein kinase (AMPK), mechanistic target of rapamycin complex 1 (mTORC1), and the NAD^+^-dependent sirtuin family (54, 55). During energy deprivation, elevated AMP/ATP ratios activate AMPK, which in turn suppresses mTORC1 by phosphorylating TSC2 and Raptor, thereby inhibiting protein synthesis and promoting autophagy (56). This ensures cellular survival under energy stress while conserving resources for essential processes.

NAD^+^ plays a pivotal role in dynamically regulating this process. Increased NAD^+^ levels activate SIRT1, which deacetylates and activates PGC-1*α*, thereby enhancing mitochondrial biogenesis and oxidative metabolism (57, 58). Conversely, NAD^+^ depletion compromises sirtuin activity, attenuating the adaptive response. Importantly, AMPK and NAD^+^ signaling are tightly coupled: AMPK enhances NAD^+^ biosynthesis through upregulation of nicotinamide phosphoribosyl transferase (NAMPT), creating a feed-forward loop that integrates energy sensing with mitochondrial function (59, 60).

This dynamic interplay exemplifies how exercise fine-tunes metabolic pathways according to energetic demands. Yet, excessive exercise may overwhelm these networks: chronic AMPK overactivation has been associated with impaired anabolic signaling and fatigue, while sustained mTORC1 inhibition can lead to muscle atrophy (61, 62). Thus, the balance between AMPK and mTORC1 is essential in defining the adaptive vs. maladaptive trajectory of exercise responses.

### 3.2 Metabolic network reprogramming and epigenetic regulation

Exercise induces systemic metabolic reprogramming, involving glucose utilization, lipid oxidation, and ketone body metabolism (63–66). For instance, β-hydroxybutyrate (BHB), a major ketone body elevated during prolonged exercise or fasting, not only serves as an alternative fuel but also functions as a signaling molecule (67, 68). BHB directly inhibits the NLRP3 inflammasome by blocking potassium efflux and preventing ASC oligomerization, thereby exerting anti-inflammatory effects (68, 69). This mechanism highlights how metabolic intermediates act as regulators of immune-inflammatory responses during exercise.

Epigenetic regulation further extends these adaptive changes. Exercise alters DNA methylation, histone modifications, and non-coding RNA expression, reshaping transcriptional programs in muscle, adipose tissue, and immune cells (69–71). For example, exercise-induced histone acetylation at metabolic gene promoters enhances oxidative capacity (71, 72), while microRNAs (miRNAs) fine-tune pathways related to angiogenesis, mitochondrial function, and inflammation (73, 74). Notably, miR-1 and miR-133a have been implicated in regulating muscle hypertrophy, while miR-21 modulates fibrosis-related signaling (75, 76). These small RNA-mediated effects, previously discussed as independent regulatory factors, are best understood within the broader context of epigenetic reprogramming, where they contribute to the persistence of exercise-induced phenotypes (77).

Taken together, metabolic network reprogramming integrates immediate energy sensing with long-term epigenetic adaptations (78). This dual regulation enables the body to flexibly respond to diverse exercise intensities. However, unresolved or maladaptive reprogramming—such as sustained inflammatory signaling or fibrosis-related gene activation—may underlie the transition from adaptive responses to pathological remodeling under conditions of excessive exercise (79, 80).

## 4 Systemic integration of exercise-induced stress responses

**Figure 3:**
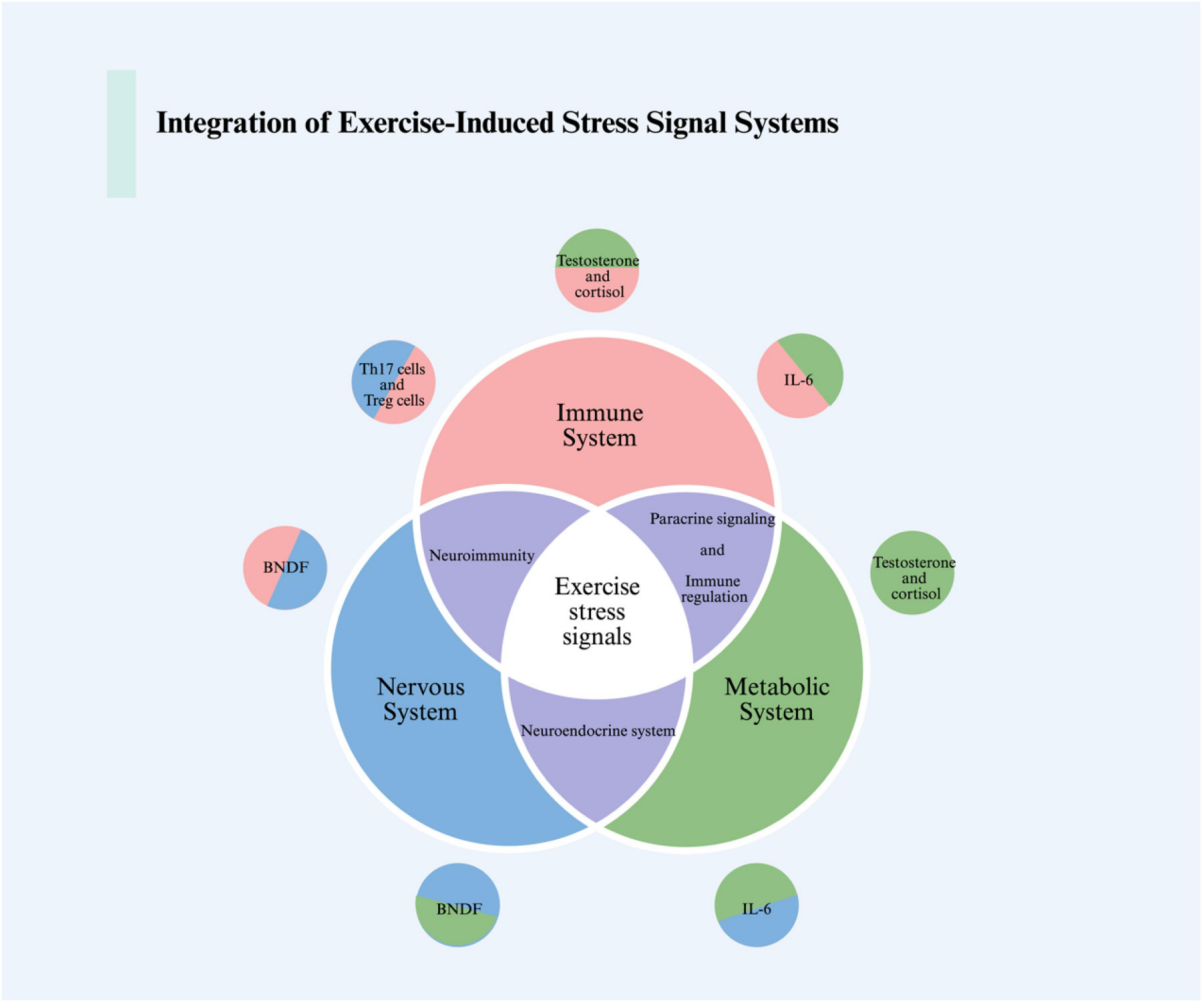
The integration of intercellular, intertissue, and intersystem communications in response to exercise stress signals is illustrated in the figure. We showcase the interactions between the nervous system, immune system, and metabolic system in response to exercise-induced stress. This includes the communication processes among immune cells, muscle cells, neurons, and how hormones and the endocrine system tie together this integrative communication process. For detailed descriptions, refer to the corresponding sections in the text. This figure was created using Biorender. BDNF, brain-derived neurotrophic factor; IL-6, interleukin-6; Th17, T helper 17; Treg, regulatory T cells.

The primary and secondary stress responses triggered by exercise ultimately converge at the systemic level, where hormones, myokines, neurotrophic factors, and immune mediators coordinate cross-tissue communication (81, 82). This integration ensures that local cellular adaptations translate into organism-wide benefits, yet it also represents the level at which excessive stress can propagate maladaptive outcomes such as chronic inflammation, neuroendocrine imbalance, or metabolic dysfunction (83, 84) (Figure 3).

### 4.1 Endocrine and myokine signaling

Exercise induces profound endocrine adjustments, including elevated catecholamines, cortisol, and growth hormone, which transiently mobilize energy reserves and promote tissue repair (85, 86). Beyond classical hormones, skeletal muscle acts as an endocrine organ by releasing myokines such as irisin, interleukin-6 (IL-6), and myostatin (81, 87). These factors exert diverse systemic effects, ranging from enhancing thermogenesis and lipid metabolism (irisin) to modulating immune cell activation (IL-6).

While moderate exercise-induced myokine release supports metabolic homeostasis and immune surveillance, excessive or chronic activation may shift this balance (5, 88). For example, persistently elevated IL-6 levels are associated with systemic inflammation and insulin resistance, whereas prolonged cortisol elevation can impair immunity and muscle protein synthesis (89). Thus, endocrine and myokine responses exemplify the bidirectional nature of exercise-induced systemic signaling.

### 4.2 Neuro-immuno-metabolic interactions: the role of BDNF

Brain-derived neurotrophic factor (BDNF) represents a critical node linking exercise-induced stress with neural plasticity and cognitive resilience. Exercise robustly enhances BDNF expression in both the hippocampus and peripheral circulation, primarily through activation of Ca^2+^-dependent CREB signaling and downstream PI3K/Akt and MAPK/ERK pathways (90, 91). BDNF binds to its receptor TrkB, promoting neuronal survival, dendritic growth, and synaptic plasticity (92, 93).

Importantly, BDNF also mediates cross-talk between the nervous, immune, and metabolic systems (94). By enhancing neuronal stress resistance, BDNF contributes to the attenuation of hypothalamic-pituitary-adrenal (HPA) axis hyperactivation, thereby reducing systemic stress hormone exposure (95, 96). Furthermore, exercise-induced BDNF upregulation has been linked to improved glucose metabolism and increased mitochondrial function in peripheral tissues, suggesting that BDNF acts as a systemic coordinator of neuro-immuno-metabolic interactions (97).

Conversely, inadequate recovery or chronic overtraining may blunt BDNF responses, impair synaptic resilience, and exacerbate neuroinflammation. Such alterations may contribute to fatigue, mood disturbances, and impaired cognitive performance commonly observed in overtrained athletes (98, 99).

### 4.3 Immune adaptation and dysregulation

Exercise exerts a dual influence on the immune system. Moderate physical activity enhances natural killer (NK) cell activity, boosts antigen presentation, and promotes anti-inflammatory cytokine profiles, thereby strengthening immune defense and surveillance against tumors and infections (100). In contrast, prolonged exhaustive exercise can suppress NK cell cytotoxicity, elevate pro-inflammatory cytokines (e.g., TNF-α, IL-1β), and increase susceptibility to infections (101–104).

At the molecular level, immune responses are tightly coupled to metabolic reprogramming. AMPK activation in T cells supports memory formation and stress tolerance, whereas excessive glycolytic reprogramming under chronic stress drives T-cell exhaustion (105, 106). This highlights the systemic feedback loop whereby metabolic and immune adaptations are intertwined in defining exercise outcomes.

## 5 Biomarkers and multimodal analyses of exercise-induced stress

Identifying reliable biomarkers and applying multimodal analytical approaches are critical for evaluating exercise-induced stress responses. Biomarkers provide measurable indicators of adaptive vs. maladaptive trajectories, while advanced analytical technologies allow for a systems-level understanding of complex responses.

### 5.1 Molecular and cellular biomarkers

Biomarkers of exercise stress span multiple categories, including mitochondrial dynamics, oxidative stress, inflammation, and cell death pathways (107). Mitochondrial fusion protein MFN2 and pyroptosis-related GSDMD have been implicated as regulators of muscular and systemic adaptation (108–111). Decreased MFN2 expression has been associated with impaired mitochondrial quality control and reduced endurance capacity (112, 113). However, current evidence is largely derived from animal and cross-sectional studies; longitudinal human cohort data are limited, and causal links to athletic performance remain speculative. Therefore, conclusions regarding MFN2 and exercise performance should be interpreted cautiously.

Oxidative stress-related biomarkers provide additional insights. Superoxide dismutase 2 (SOD2), glutathione peroxidase (GPx), and catalase represent key antioxidant defenses upregulated during moderate exercise (112, 114, 115). Conversely, excessive or exhaustive exercise often leads to their depletion alongside increased lipid peroxidation (MDA) and elevated pro-inflammatory cytokines such as TNF-α and IL-6 (116). These markers not only indicate cellular redox balance but also reflect systemic inflammation, making them valuable for assessing the transition from physiological adaptation to pathological stress.

### 5.2 Epigenetic and non-coding RNA biomarkers

Exercise alters the expression of various non-coding RNAs, which can serve as potential biomarkers of adaptive remodeling or pathological stress. For example, miR-1, miR-133a, and miR-206 are strongly linked to muscle hypertrophy and regeneration (117–119). In addition, miR-29b has been reported to inhibit fibrosis in certain experimental settings (120, 121). However, some studies—such as the use of nanoparticle-delivered miR-29b to inhibit fibrosis—were conducted *in vitro* under osteogenic conditions rather than in the context of exercise-induced cardiac fibrosis (122, 123). This discrepancy highlights the importance of contextual validation before extrapolating findings to exercise physiology.

### 5.3 Multimodal analytical approaches

Advances in high-throughput and single-cell technologies enable a multimodal perspective on exercise-induced stress (124). Single-cell transcriptomics, proteomics, and metabolomics provide unprecedented resolution in capturing cell-type specific responses (125). For example, single-cell sequencing has revealed exercise-induced heterogeneity in immune cell metabolic reprogramming (126). Moreover, extracellular vesicles (EVs), including exosomes, have gained attention as carriers of exercise-induced signals (127, 128). Reports suggest that EVs can transport transcriptional regulators such as PGC-1*α* mRNA, thereby influencing mitochondrial biogenesis (129, 130). However, most current evidence stems from neural stem cell-derived exosome studies rather than direct exercise experiments, and the causal relationship between exercise, exosomal cargo, and enhanced endurance capacity remains to be clarified (129, 131).

Therefore, while exosomes and other multimodal biomarkers hold great promise, more rigorous exercise-specific experimental validation is needed to confirm their functional relevance.

### 5.4 Integrative framework and limitations

Multimodal biomarker approaches must account for inter-individual variability, including sex, age, genetic background, and training status (132). These factors can significantly modulate biomarker responses, complicating the definition of universal thresholds. For example, older individuals may exhibit blunted antioxidant responses (133), while genetic polymorphisms in mitochondrial genes could influence stress resilience. Integrating multimodal datasets with clinical phenotypes is thus essential to establish robust biomarkers for guiding personalized exercise prescriptions.

## 6 Discussion and future directions

This review has summarized how exercise-induced stress responses progress from primary mechanical and organelle signals to secondary metabolic regulation and ultimately to systemic integration across endocrine, immune, and neural networks. By organizing these responses into a layered cascade—primary responses, secondary regulation, and systemic integration—we have highlighted the dual nature of exercise as both a health-promoting and potentially pathological stimulus. A central theme emphasized throughout this review is the bidirectional threshold theory, which provides a conceptual framework for understanding how exercise intensity and duration determine biological outcomes. While moderate exercise promotes beneficial adaptations such as mitochondrial biogenesis, enhanced antioxidant defense, and improved neuroplasticity, excessive or prolonged exercise can lead to maladaptive processes including calcium overload, mitochondrial permeability transition pore (MPTP) opening, maladaptive ER stress, chronic inflammation, and fibrosis (134, 135). However, a key limitation of the current literature is the imbalance in mechanistic evidence: adaptive responses are well characterized, but the molecular underpinnings of maladaptive trajectories remain less systematically explored. For example, while Piezo1 activation is known to facilitate vascular remodeling, its potential contribution to pathological calcium influx and tissue fibrosis under sustained activation has not been rigorously studied (136, 137). Similarly, the transition from adaptive unfolded protein response (UPR) to pro-apoptotic ER stress during exhaustive exercise requires more *in vivo* validation.

Another limitation lies in the translation of experimental findings to human physiology. Much of the mechanistic data derives from animal models or *in vitro* systems, which may not fully capture the complexity of human exercise responses. Longitudinal human cohort studies are scarce, making it difficult to establish causal links between molecular markers (e.g., MFN2, SOD2, exosomal cargo) and real-world exercise outcomes such as performance, recovery, and disease risk. Moreover, individual differences—including sex, age, training history, and genetic background—are seldom addressed in mechanistic studies, yet they critically shape exercise-induced stress responses.

While exercise is broadly beneficial, the potential for maladaptation or pathological damage cannot be overlooked, particularly in high-intensity or prolonged regimens. A balanced perspective requires integrating monitoring tools that can detect when beneficial adaptation shifts toward risk. Practical approaches include setting training intensity using relative measures such as %VO_2_max or %heart rate reserve (HRR) (138), tracking recovery via heart rate variability (HRV) and lactate clearance, and assessing biochemical markers such as creatine kinase (CK), interleukin-6 (IL-6), and oxidative stress indices (139–141). In addition, validated psychometric tools (e.g., RESTQ-Sport, Profile of Mood States) can identify early warning signs of overreaching or overtraining (142). These approaches should be viewed as pragmatic starting points rather than definitive guidelines. Further longitudinal clinical studies are required to validate and standardize risk-stratification strategies for different populations.

An important limitation of the bidirectional threshold framework is its sensitivity to individual-specific factors. Ageing is associated with reduced mitochondrial adaptability and a blunted antioxidant response, lowering the threshold at which maladaptive effects emerge (143, 144). Sex and hormonal status, particularly estrogen levels, modulate inflammatory and oxidative stress pathways, contributing to sex-based differences in training outcomes (145). Genetic background (e.g., polymorphisms in ACTN3, PGC-1α) further influences cardiorespiratory fitness and muscle adaptation (146). Training history also determines baseline resilience: well-trained individuals often exhibit attenuated biomarker responses compared with untrained individuals under the same workload. Finally, comorbid conditions such as diabetes, obesity, or cardiovascular disease substantially modify exercise-induced stress responses, often lowering tolerance and increasing risk for maladaptation. Collectively, these factors underscore that the “bidirectional threshold” must be interpreted flexibly rather than as a universal cut-off, highlighting the need for personalized approaches in both research and clinical translation.

From a methodological perspective, the integration of multimodal omics technologies (e.g., single-cell transcriptomics, proteomics, metabolomics) with clinical phenotyping offers a promising avenue to bridge mechanistic insights with human variability. However, technical challenges remain, such as harmonizing data across platforms, capturing transient exercise responses in real time, and distinguishing adaptive vs. maladaptive signatures within heterogeneous cell populations.

Looking forward, several areas warrant particular attention:

1. Defining molecular thresholds of adaptation vs. maladaptation across exercise intensities and modes (endurance vs. resistance), with quantitative markers to guide individualized exercise prescriptions.
2. Mechanistic studies of maladaptation, including Piezo1-mediated calcium overload, chronic FAK signaling, MPTP dysregulation, and maladaptive ER stress.
3. Validation of biomarkers in human cohorts, with longitudinal tracking to establish predictive value for performance, recovery, and disease outcomes.
4. Integration of multimodal datasets to capture the systemic nature of exercise responses, with a focus on linking molecular pathways to functional outcomes.
5. Personalized exercise medicine, leveraging genetic, epigenetic, and metabolic profiling to design tailored interventions that maximize benefits while minimizing risks.

In conclusion, the biological responses to exercise stress are not unidirectional but exist along a continuum shaped by intensity, duration, and individual context. By advancing our understanding of both adaptive and maladaptive pathways, future research can refine exercise as a precise therapeutic modality—balancing health promotion with the prevention of overtraining-related pathology.
